# Preparation of humic acid-bentonite polymer composite: A heavy metal ion adsorbent

**DOI:** 10.1016/j.heliyon.2022.e09720

**Published:** 2022-06-20

**Authors:** Evelina L.M. Amutenya, Fengshan Zhou, Jinliang Liu, Wenjun Long, Liang Ma, Meng Liu, Guocheng Lv

**Affiliations:** Beijing Key Laboratory of Materials Utilization of Nonmetallic Minerals and Solid Wastes, National Laboratory of Mineral Materials, School of Materials Science and Technology, China University of Geosciences (Beijing), No. 29 Xueyuan Road, Haidian District, Beijing, 100083, PR China

**Keywords:** Bentonite, Humic acid, Polymer, Adsorption properties, Heavy metal ions, Wastewater

## Abstract

Adsorbents for wastewater treatment have evolved from scientific adsorbents to natural adsorbents. In this study, a humic acid-bentonite polymer (HBP) composite comprising humic acid, bentonite, and anionic polyacrylamide was integrated into an anionic polyacrylamide (aPAM) polymer matrix as an adsorbent pellet with N, N-methylene-bis-acrylamide (MBA), sodium tetraborate pentahydrate and chromium chloride is used as a novel adsorbent to remove Ni^2+^, Cd^2+^ and Pb^2+^ ions from aqueous solution. Sorption of these ions onto HBP is studied as a function of pH, adsorbent dosage, initial metal ion concentration, humic acid, and bentonite properties to evaluate adsorption efficiency. The results showed that adsorption sharply depends on pH, metal ion concentration, and contact time, but is complemented by humic acid and bentonite properties. The adsorption increased from 8% to 94.7% in the first 30 min at respective pH values of 5.6 and 9 for (Ni^2+^, Cd^2+^, and Pb^2+^). The HBP sorption power decreased with increasing adsorbent dosage, while the adsorption efficiency increased in ascending order for the cations Pb^2+^, Ni^2+^ and Cd^2+^ with efficiencies up to 94.7%, 90.9%, and 90.2%. The experimental data for Ni^2+^, Pb^2+^ adsorption fits the Langmuir isotherm, while that for Cd^2+^ adsorption fits the Freundlich isotherm. HBP showed modest adsorption performance at low and high concentrations, this is attributed in large part to the humic acid and bentonite properties that affect HBP's unique performance.

## Highlights

• A novel composite material prepared by coupling HA, aPAM, and Bentonite properties.
• Composite adsorption properties efficient than clay and polymer individually.
• Humic acid cationic adsorbing properties increased the uptake of heavy metal ion.
• Sodium bentonite isomorphous substitution compensates HBP cat-ion exchanging abilities.
• HBP is efficient and eco-friendly for industrial wastewater treatment.

## Graphical abstract

**Figure:**
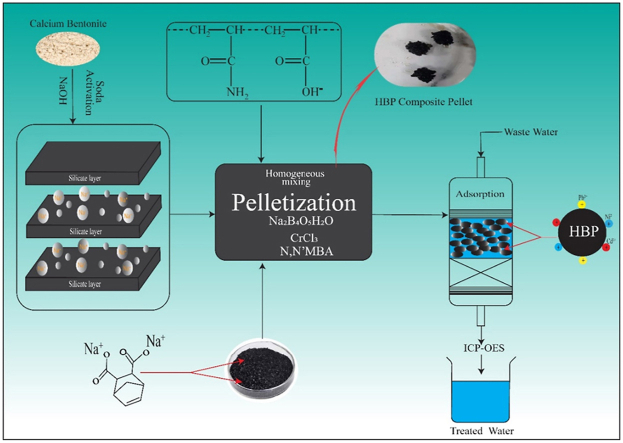

## 1 Introduction

Wastewater-related discharges originating from various processes involving technical developments pose a high risk to human health and the environment if not treated or disposed of safely. Therefore, regulations are put in place to minimize human and environmental exposure to heavy metals and other hazardous chemicals. The regulations include limits set on various types as well as their permissible concentrations present in the discharged wastewater [1]. Among the problems posed by the unsafe disposal of untreated wastewater into the environment is the leaching of excess heavy metal ions, fluoride, arsenic, and organic matter into drinking water streams, resulting in degraded water quality and polluted soils. Human exposure to heavy metal ions leads to various human diseases, such as respiratory problems, kidney diseases, neurological disorders, and cancer. Although it is scientifically impossible to completely prevent the leaching of heavy metal ions into the environment, the elimination of heavy metals from wastewater has been accomplished by using polymer materials and clay minerals. However, it is currently being dominated by the use of low-cost natural adsorbents, such as bentonite clay and humic acid which are non-toxic to the ecosystem [2].

Bentonite, a layered aluminum silicate clay composed of montmorillonite and related clay minerals of the smectite group, is characterized by its extensive water adsorption properties due to a high surface area per unit weight and its ability to base exchange due to its typical 2:1 layered silicate structure and has been used for decades as natural or modified. The role played by clay minerals in environmental protection is owed to their cation exchange capacity in transportation of toxic materials [3]. In this sense, the adsorption of heavy metals by clay minerals is quite well documented in the specialized works of literature [4, 5, 6].

Bentonite-based adsorbents differ significantly from other adsorbents because the clay structure consists of two tetrahedral silica layers composed of 4 hydroxyl groups surrounding each silicon atom in a tetrahedral arrangement. It is this layered silicate structure that contributes to its large surface area per unit weight, resulting in its interlayer gap being between 10 and 15 Å, giving the clay its property of exchangeable cation capability in its interlayer space. These silica layers possess a slight negative charge effect, that is counteracted for by exchangeable cations in the interlayers [7]. This particular charge happens to be very weak causing cations such as Na^2+^ to be adsorbed with an associated hydration shell, and when these cations are held in this pattern, in a bentonite clay interlayer, they can be easily replaced by ion exchange [7].

With activation and modification processes, the properties of the clay surface such as pore volume, surface area, and surface acid sites are improved, giving the edges of the clay surface as well as the surface of its particles the ability to adsorb anions, cations, and non-polar ionic impurities, because it is now more organophilic or hydrophilic. When bentonite is modified, the clay and its composites become particularly effective in adsorption of heavy metals and have received a vast and excellent attention as adsorbents for metals such as mercury [5], copper [8], nickel and cadmium [9], zinc, manganese, cobalt, and nickel [4], chromium [10], cadmium and mercury [11].

On the other hand, chelates such as humic acids (HA) are currently the most promising green biomaterials for wastewater treatment, which have a strong ability to sorb cationic and hydrophobic organic pollutants [12]. The humate group used in this study is the carbohydrate group or sugars, and it contains a variety of active groups such as carboxyl groups, phenolic hydroxyl groups, carbonyl groups, sulfonic acid groups, and methoxy groups, which have an impact on important adsorption influencing properties such as the acidity, ion exchange, colloidal, and complexation properties [13]. This function is what gives HA a potential to purify metal ions and enhance water quality [14, 15], all in attribution to the fact that they form quite stable complexes upon interactions with heavy metal ions [16].

Although polymeric materials have represented a major development in adsorption processes for treating wastewater, currently clay-polymer composites or modified clay and humic acid composites effectively treat both inorganic and organic micropollutants in wastewater, but these adsorbents and their composites have disadvantages such as high cost and inability to effectively remove heavy metal ions at low concentrations. The outermost short come modelled by most heavy metal ion adsorbents is the materials used in synthesizing these adsorbents, and this tends to heavily affect dependent variables in the adsorption process such as the adsorption capacity of the adsorbent toward the targeted contaminant, cost/efficiency ratio, and the type and concentration of the contaminants present in water [13]. The adsorbents considered convenient must be available, of low cost, easily regenerable with high selectivity toward targeted contaminants [17]. Interestingly, the adsorption method effluents must as well produce low quantities of sludge [17].

Efforts to create new adsorbents have highlighted these developments and made it necessary to renew state-of-the-art adsorbents synthesized from natural adsorbents to improve adsorption capacities at both low and high metal ion concentrations. A key issue in this context is that these new materials may exhibit more properties of an effective adsorbent than polymer, humic acid, and clay minerals individually, making them more preferable [18] In addition, an ideal adsorbent is expected to be readily available, and have sufficient adsorption capacity as well as selectivity under given operating conditions. Hence, the coupling of structural properties and the modification of material properties in nanotechnology has led to the birth of many innovative functional materials.

In this study, a novel and inexpensive HBP adsorbent is developed from a homogeneous mixture of activated sodium bentonite clay and sodium humate, followed by polymerization using a water-soluble polymer, aPAM. Therefore, for the adsorbent properties to be intrinsically allied to the design of the fixed-bed column adsorption process, the polymerized humic acid-bentonite mixture is pelletized into small cylindrical pellets using a cross-linker N, N-methylene bisacrylamide (MBA), binder; Sodium tetraborate pentahydrate and chromium chloride. Applying this coupling technique, the resulting composite produces more adsorption sites for heavy metal ions due to the ion exchange capacity of bentonite clays and high affinity for heavy metal ions, due to the adsorption effect of humic acid cations, resulting in an adsorbent with an increased ability of cationic and hydrophobic sorption of organic pollutants and last but not least an effective adsorbent.

The modified composite demonstrated potential universal ion exchange capabilities with far longer adsorption time before adsorbent saturation in comparison to other composites, forming complexes with metal ions and providing a broad spectrum as an adsorbent system. HBP's sorption capacity was examined against Freundlich and Langmuir isotherms, leading to favorable results. The synthesis of HBP is inexpensive and can be carried out by an appropriate pelletization process in the absence of heat provided that the process values are consistent with findings of the content of both bentonite and humic acid to provide the composite's ability to remove heavy metal ions, adsorption properties, adsorption capacity, and efficiency. However, further research is needed here to investigate the desorption of HBP, regeneration, and reusability of the adsorbent.

### 1.1 Adsorption kinetics

An adsorption isotherm is required to provide data on the limit of the extent at which a solute is adsorbed by an adsorbent from a liquid at a constant temperature [19]. Adsorption of solutes is accomplished by physical adsorption, chemical adsorption, or ion exchange [19]. To predict the precise mechanism on how the adsorption process will be is quite complex as it depends on the characteristics of the solute as well the adsorbent [19]. To model equilibrium data, the Langmuir model is used with its conclusions based on assuming that a monolayer adsorption takes place on a structurally homogeneous surface, and there is no interaction between molecules adsorbed on neighboring sites whatsoever [18]. The linear form of the Langmuir isotherm is given by Eq. (1):

(1) $$\frac{\text{C}e}{\text{q}e}=\frac{1}{\;\text{qmax}\cdot K_{L}}+\frac{Ce}{\text{qmax}}$$

where ${\text{q}}_{\text{e}}\text{ ​}\left({\text{mg·g}}^{-1}\right)$ is the sorbate amount adsorbed onto HBP at equilibrium, $K_{L}\left({\text{L·mg}}^{-1}\right)\text{ ​}$ is Langmuir equilibrium constant, and q_max_ (mg·g^−1^) is the maximum amount of heavy metal ions per unit weight of HBP adsorbent to successfully reach a complete monolayer coverage capacity [18]. The Langmuir constants $\left(K_{L}\text{ ​and ​}{\text{q}}_{\text{max}}\right)$ are be determined from the plot of $C_{e}\text{/}q_{e}$ versus $C_{e}\text{.}K_{L}$ related with the evident sorption energy and q_max_ to reflects a full monolayer (mg·g^−1^) [18]. As shown in Eq. (2), the vital attributes of Langmuir can be described as a dimensionless constant separation factor or equilibrium parameter, *R*_*L*_ [18]*,* which is expressed by the value of R, given by the formula:

(2) $$R_{L}=\frac{1}{1+KL\;Co}\;$$

The value of $R_{L}$ indicates the shape of the isotherm which is unfavorable $R_{L}$ (>1), linear ($R_{L}$ = 1), favorable (0 < $R_{L}$ <1), or irreversible ($R_{L}$ = 0) [18]. On the other hand, the Freundlich isotherm model represents the multilayer adsorption on heterogeneous surfaces where the interactions between adsorbed molecules are observed [18], expressed according to Eq. (3) as follows:

(3) $$\text{log}\left(qe\right)=\log\left({{\text{K}}_{\text{f}}}^{\text{O}}\right)+\frac{1}{n}\text{log}\left(Ce\right)$$

where ${\text{K}}_{\text{f}}({\text{mg}}^{-1-1\text{/n}})\text{ ​}{\text{L}}^{1}{\text{/ng}}^{-1})$ is the Freundlich constant that represents the parameter characterizing quasi-Gaussian energetic heterogeneity of the adsorption surface [18]. The ${\text{K}}_{\text{f}}$ value is interrelated to the adsorption capacity while the slope 1/n, ranging between 0 and 1 evaluates the adsorption intensity which becomes more heterogeneous as it approaches zero [18]. Nevertheless, values below unity suggests a chemisorption process where 1/n above 1 is indicative of cooperative adsorption [18].

Linear regression (*R*^*2*^) is very crucial designing liquid-phase adsorption systems, hence to determine the best model fitting a certain adsorbent, *R*^*2*^ is used as an indicator, however its usage is limited to solving isotherm models that present linear forms [20]. Hence the average relative error (R_*L*_) is employed to fit isothermal equations to the experimental data obtained [18]. The constants and correlation coefficients of Pb^2+^, Cd^2+^, and Ni^2+^ cations adsorption by HBP are presented in Table 4.

## 2 Experimental setup

### 2.1 Materials and reagents

The materials used in this study are modified sodium humate, modified sodium bentonite, and anionic polyacrylamide. Raw natural calcium bentonite was obtained from Inner Mongolia Autonomous Region, China, which was activated and used as the raw material for the adsorbent preparation. The clay was whitish and received in a fine powder form, approximately 100 μ. Modified sodium humate was obtained from Wuhai and Erdos of Inner Mongolia and finely ground to obtain 100 μ with a high-speed mixer to facilitate homogeneous mixing. Anhydrous sodium carbonate, methylene blue, chromium chloride, sodium borate-sulfuric acid diluted to 38%, and sodium hydroxide were purchased from Sinopharm Chemical Reagent Beijing Co., Ltd. All of which are chemical grade (CP). A crosslinking Agent, NN-methylene-bisacrylamide, and linear anionic polyacrylamide were purchased from Beijing Heke Polymer Material Co. Ltd. Syringe filters (0.22 m) were obtained from Haining De Filter New Material Technology Co. Ltd. Standard solutions of 1000 μg·ml^−1^ concentration of Ni^2+^, Cr^2+^, and Pb^2+^ were purchased from Beijing Haohan Spectrum Technology Co and sterile syringe filters (0.22 μm) were purchased from Haining De Filter New Material Technology Co. Ltd.

### 2.2 Methods

#### 2.2.1 Equipment and accessories

The instruments and devices used in the experiment are the electronic balance (accuracy in 0.01 g) for weighing samples, a high-speed mixer; to evenly disperse the clay and sodium carbonate in deionized water before activation, shake water bath incubator, graduated cylinder, syringe, beaker, spatula, oven, high-speed homogenizer, and a centrifuge. The X-ray diffractometer (XRD) device, model Rigaku D/max, was used to test the mineral composition of bentonite and sodium humate with Cu K (λ = 0.15406 nm) radiation; X-ray fluorescence (XRF) equipment, model Rigaku ZSX Primus, was used to determine the elemental composition of bentonite, with sodium being the initial determining element. The surface morphology of HBP was characterized using the scanning electron microscope (SEM) (SU8020, Hitachi; Tokyo, Japan) operated at an accelerating voltage of 5 kV to examine and analyze the surface morphology and provide evidence of bentonite, polymer, and humic acid present in the composite and effects of heavy metal cations after adsorption. The surface functional groups of HB (mixture of humic acid and bentonite) were characterized by Fourier transform infrared spectroscopy (FTIR). The IR wavelength was run between 4000 and 500 cm^−1^ operating at a peak resolution of 4 cm^−1^.

The American Inductively Coupled Plasma-Optical Emission Spectroscopy, Agilent (ICP-OES 730) was used to determine the residual metal ion concentration after adsorption. Microscale observations and chemical composition analyses of the reaction products were performed using a Field Emission Scanning Electron Microscope (SEM) equipped with Energy Dispersive Spectrometry (EDS), model HORIBA EX 350i. The XRF results of calcium bentonite and activated sodium bentonite are presented in Table 1.

**Table 1:** XRF analysis results of calcium-bentonite and Activated Sodium-bentonite samples (wt.%).

Chemical compound	Calcium bentonite (raw)	Sodium bentonite (activated)
SiO_2_	55.00	51.38
Al_2_O_3_	16.19	13.04
Fe_2_O_3_	7.66	8.15
Cao	3.98	3.84
MgO	3.92	3.29
Na_2_O	0.189	1.70
K_2_O	0.367	0.438
TiO_2_	0.689	0.406
SrO	0.250	0.131

### 2.3 Theory and sample preparation

#### 2.3.1 Modification of calcium bentonite to sodium bentonite

Calcium bentonite was modified by the soda activation process by stirring 25% by weight clay in 500 ml deionized water at a speed of 10,000 rpm for 10 min with a high-speed homogenizer. A 4% by weight amount of sodium carbonate was weighed and added to the mixture, which was again stirred for 30 min with a high-speed homogenizer at 3000 rpm. As suggested by Magzoub [21], the suspension was transferred to a magnetic stirrer for 24 h at a temperature (*T*) above 60 °C and the resulting modified slurry was centrifuged at 10,000 rpm for 10 min, after which the top part was removed and has been dried as the purified sodium bentonite leaving a precipitate of impurities at the bottom. The activated sodium bentonite was dried at 75 °C for 6 h, ground to a fine powder (100 μ) for experimental use, and designated Na-BT. Calcium bentonite (Ca-BT) is a useful adsorbent for ions in solution, but when converted to sodium bentonite it tends to exhibit many of the properties of sodium bentonite via the ion exchange process, making it particularly useful in the adsorption process [2].

**Figure 1:**
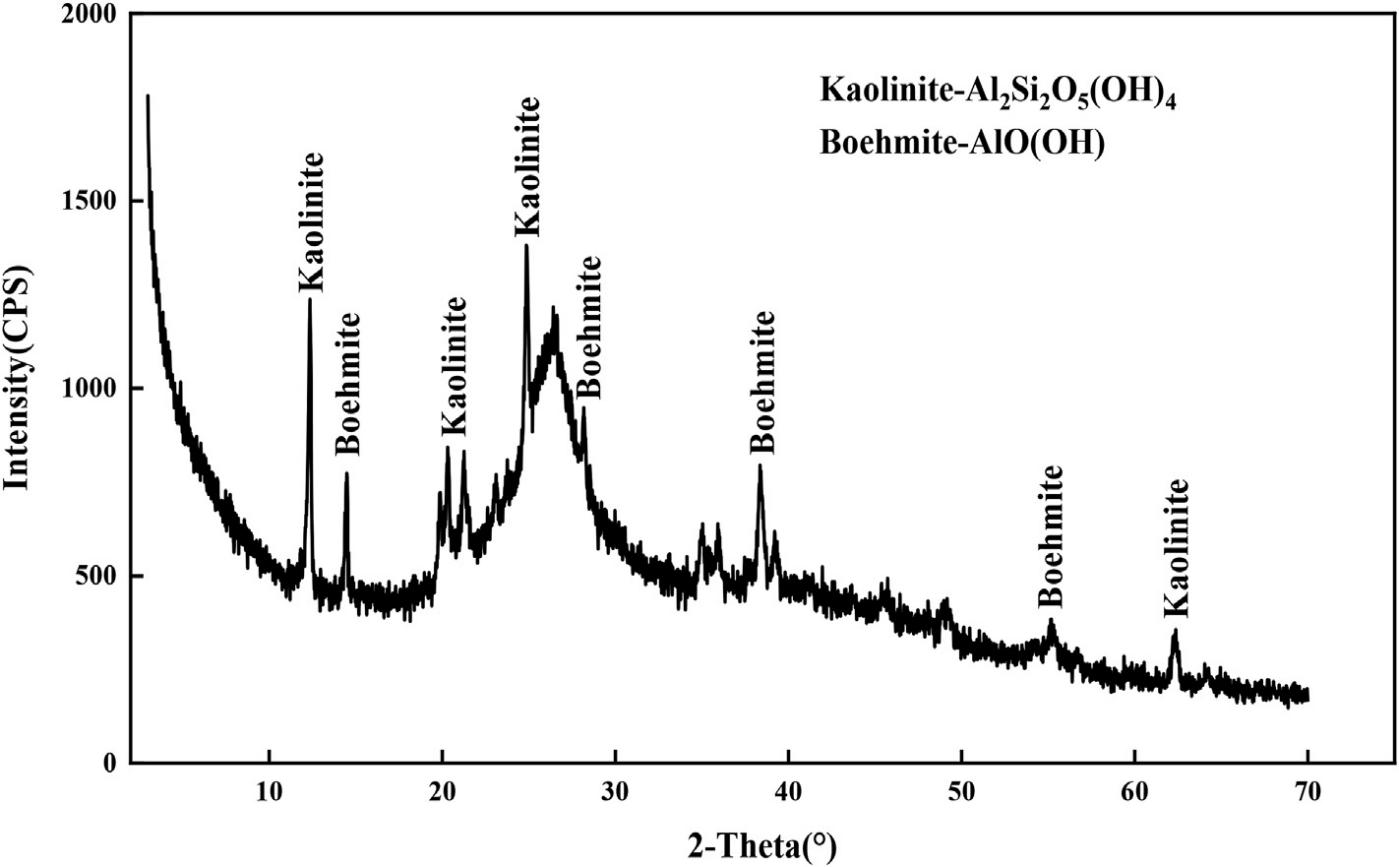
XRD patterns of sodium humate.

**Figure 2:**
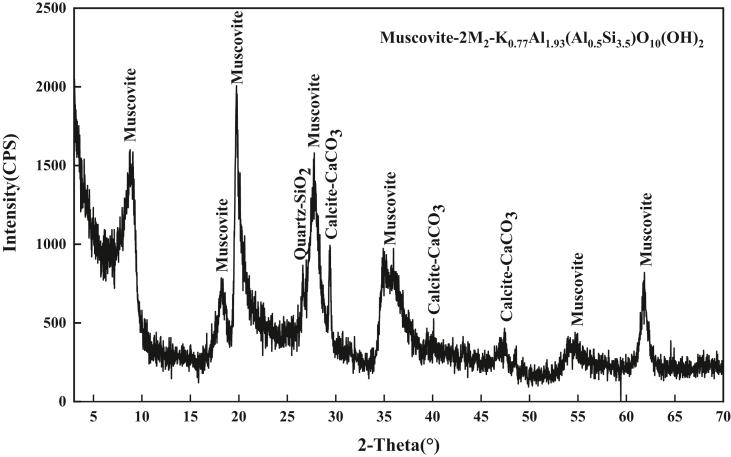
XRD patterns of activated sodium bentonite.

After soda activation, a decrease in SiO_2_ and Al_2_O_3_ was observed, confirming the removal of impurities such as quartz. As shown by the XRD and XRF results in Table 1 and Figure 1, calcite was present in Ca-BT and the amount of calcite identified after activation reflects both the interlayer and the calcite content of the clay. The sodium content of Na-BT has increased compared to that of Ca-BT and this only reflects the Na^+^ interlayer cations after soda activation which is due to the conversion of Ca-BT to Na-BT, with sodium being the dominant exchangeable cation is of activated clay. In Figure 2, XRD diffraction patterns representing the mineralogical analyses of sodium humate used in this study is presented.

#### 2.3.2 Evaluation for Na-BT cation exchange capacity

The Na-BT cation exchange capacity was determined by the Methylene blue test as described by Hayati-Ashtiani [22]. The procedure for calculating the cation exchange capacity (CEC) of bentonite is calculated by the formula given in Eq. (4):

(4) $$CEC\left(\frac{mEq}{100g}\right)=\frac{\left(MB\;added\;\left(mL\right)\;M\;\left(g\right)\right)}{Volume\;of\;MB\;solution\left(cc\right)}$$

where MB is the amount of methylene blue solution used to reach the endpoint of the test while M is the dry weight (g) of methylene blue and V is the volume of MB solution (mL) used. The Cation Adsorption Capacity (CEC) test results for raw Ca-BT and activated Na-BT obtained were 64 meq/100 and 82 meq/100.

#### 2.3.3 Preparation of bentonite-humic acid mixture (HB)

25 g humic acid with a particle size of 200 is weighed and subjected to size reduction passing 200 in a high-speed milling mixer. Another 25 g of Na-BT clay with a particle size of 100 was weighed out and the two ingredients were proportioned to the desired ratios (Hm: Bent (1:1; 4:2; 7:3; 2:4; 3:7) in a high-speed mixer at 300 rpm for 2–3 min to obtain a blended homogeneous mixture, resulting in 5 samples labeled HB1, HB2, HB3, HB4, and HB5 presented in Table 2.

**Table 2:** Equilibrium studies of Pb^2+^ adsorption by HBP at a concentration (*I*) of 5 mg. L^−1^, an adsorbent dosage of 1 g/100 mL, temperature (*T*) of 25 °C, and a time (*t*) period of 24 h.

Sample	Bent: Hm (%)	aPAM (%)	Pb^2+^ removal (%)	q_e_ (mg·g^−1^)	C_t_ (mg·L^−1^)
HBP_1_	50:50	15	46.00	2.700	0.23
HBP_2_	60:40	15	38.60	0.193	3.07
HBP_3_	70:30	15	34.40	0.172	3.28
HBP_4_	40; 60	15	79.60	0.398	1.02
HBP_5_	30:70	15	91. 38	0.457	0.43

**Figure 3:**
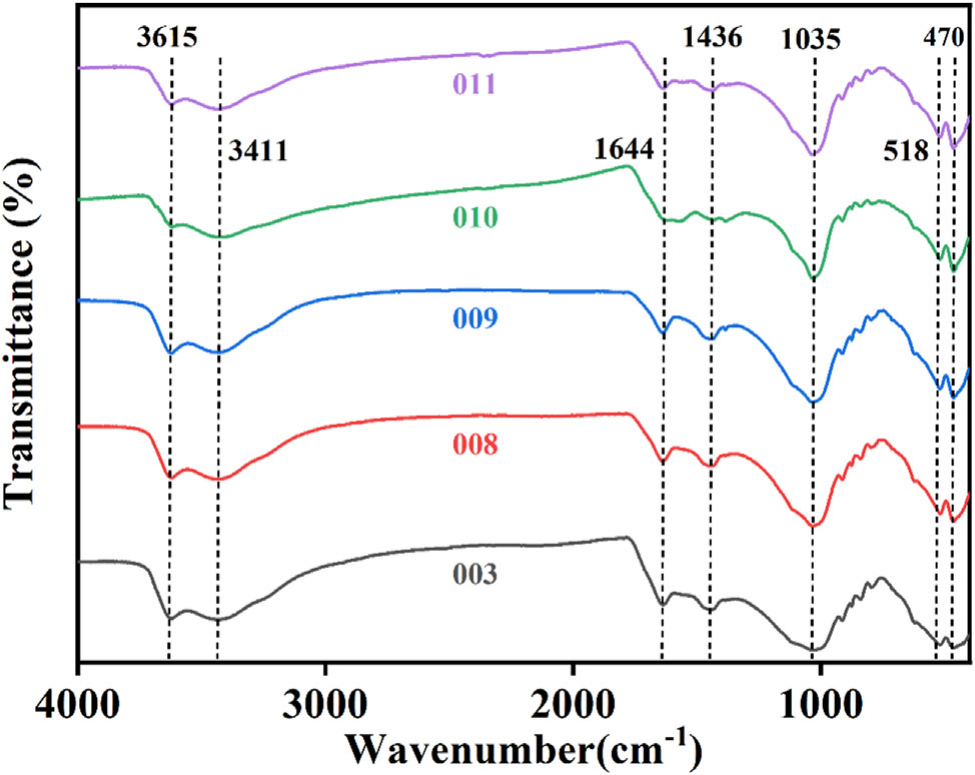
FTIR spectra of HBP_5_.

As shown by Eq. (5), the amount of heavy metal ions adsorbed onto HBP (q*e*) is calculated by a mass balance equation of heavy metal ions before and after adsorption:

(5) $$qe=\frac{\left(Co-Ct\right)}{M}\times V$$

where, *q*_*e*_ is the amount of Pb^2+^ adsorbed; (mg·g^−1^); *M* = mass of the adsorbent, (g), and *V* is the volume of adsorbate solution (L) [20]. The sample HB5 is therefore selected as a raw material suitable for the synthesis of an experimental adsorbent during this study as it was decided that it provides better adsorption results. The IR spectra of HB5 were obtained by Fourier infrared spectroscopy (FTIR) before performing the palletizing process and the characterization results are presented in Figure 3.

#### 2.3.4 Preparation of (HBP) adsorbent pellets

To prepare HBP adsorbent pellets, the procedure contained in US Patent No. 19,1992 was followed. The selected HB5 is polymerized using aPAM in weight percentages ranging from 85% HB5 to 15% aPAM, respectively. The weight percentages are based on the total of the two components, which in this study was 4.25 g, followed by the addition of deionized water and stirring to a homogeneous mixture and aging for 15 min. The amount of water required is 10–40% by weight, depending on the amount of HB5 and aPAM in the feed. The crosslinking agent MBA was then added in an amount of less than 4% by weight, which in this case was 0.25 g, depending on the weight of aPAM in the feed. The crosslinking agent MBA was then added in an amount of less than 4% by weight, which in this case was 0.25 g, depending on the weight of aPAM in the feed.

After successful polymerization of HB5, the sample is subjected to an aging period of 35 min, the granulation of which begins with the addition of 1 g sodium tetraborate pentahydrate to promote adhesion and 0.25 g chromium chloride as a binder to improve the mechanical properties of the pellets. The amount of both sodium tetraborate pentahydrate and chromium chloride varies from 1-2% by weight and their weight depends on the weight of aPAM in the feed. The blend composition was transferred to a barrel and cylindrically shaped pellets of varying sizes were extruded, however, their diameter ranged from 0.125 inches to not more than 0.25 inches, while the length was from about 0.25 inches to about 0.75 inches. The extruded pellets were dried at room temperature without heat, because exposure to heat leads to carbonization of the humic acid in the mixture, causing the humic acid to lose its cationic adsorption activity as observed in studies by Aftab [23] on complexing properties of humic acids with metal cations, where he concluded poor adsorption as a function of lower levels of (C/H) ratios during insolubilization of HA by heating, resulting in different acid groups present on HA affecting the metal complexation constant of acid groups; thereby reducing to some extent the number of available metal-binding sites on the immobilized HA. He further suggested that the reduction in solubility might be attributed to a partial loss of COOH and OH groups. The weight of the wet and dry pellets was recorded before drying to determine the water content [24].

#### 2.3.5 The density determination and water resistance of pellets

According to the European Committee for Standardization (CEN), bulk density is a meaningful pellet specification, as is moisture content and swelling index [25].

• *Density*

The density of HBP pellets was determined using the methods discussed by Jaya [25]. Dry pellets were weighed before and after immersion in a graduated cylinder containing 100 mL of distilled water at its initial pre-immersion volume (V_*0*_), which caused the graduated cylinder reading to change to V_*1*_ after the pellet had been in place for some time, approximately 20 min. The process was performed in triplicate with different pellets and the value of V1 is taken as the average of the 3 cycles [25]. The results are shown in Table 3.

**Table 3:** Water content and density analysis for HBP pellets.

Sample	Water content analysis			Density analysis for HBP pellets		
	W_c_ (g)	W_d_ (g)	W_i_ (%)	M_d_ (g)	V_f_ (L)	Density (g·L^−1^)
1	0.72	0.67	6.90	0.62	104.30	0.594
2	0.82	0.76	13.25	0.73	107.25	0.680
3	0.68	0.59	13.23	0.69	107.20	0.643
**Av.**	**11.4%**			**0.644**		

Density is therefore calculated by the formula given by Eq. (6):

(6) $$\text{ρ}=\frac{\text{M}}{\text{V}0-\text{ ​V}1}$$

where, ρ is density, g.cm^3^; V_0_ is the initial volume before introducing the pellet (mL) and M is the mass of dry pellet (g) and V_1_ represents the final volume after introducing the pellet mL [25].

• *Water content*

To determine the water content of the pellets (W*i*), dry pellets of known mass (W_d_) were immersed in distilled water for 30 min and the weight (W_c_) was recorded thereafter. The water content is the percentage weight gain (%) relative to the initial pellet weight calculated using Eq. (7):

(7) $$\text{W}i=\frac{\text{Wd ​}-\text{ ​Wc}}{\text{Wc}}\text{∗}100$$

where W_*i*_ is the water content, (%); W_d_ is the dry pellet weight (g) and W_c_ is the wet pellet weight (g). The tests were run in triplicate and the values of W_d_ and W_c_ are the mean of the 3 cycles shown in Table 3.

#### 2.3.6 Synthesizing aqueous heavy metal ions solution

The synthesized heavy metal-containing aqueous solution is made using 3 of the selected heavy metals (Ni^2+^, Cr^2+^, and Pb^2+^). All solutions were made up of deionized water and diluted according to the desired concentrations.

### 2.4 Experimental procedures

#### 2.4.1 Equilibrium studies

Sorption of Ni^2+^, Cr^2+^, and Pb^2+^ cations was performed using the batch experiment technique of single solute reactions. The experiments were performed in 100 ml glass columns with stoppers containing 5 mg·L^−1^ aqueous solutions of Pb^2+^, Ni^2+,^ and Cd^2+^ with 1 g HBP. The adsorbent and aqueous solution were shaken at 200 rpm to reach equilibrium concentration while the columns were kept sealed to minimize losses to the atmosphere as per procedures by [18]. Samples were given a reaction time of 24 h at 25 ± 2 °C, and after the reaction time, the endpoint sample is collected and filtered through a 0.2045 μm membrane filter and analyzed for heavy metal ions by ICP-OES. The results obtained are shown in Table 2.

#### 2.4.2 Effect of solution pH

Aqueous solutions of Pb^2+^, Ni^2+,^ and Cd^2+^ with an initial metal concentration of 5 mg·L^−1^ was used and the effect of solution pH was studied by varying the initial pH over the range 2–10 while giving a reaction time of 24 h at 25 ± 2 °C. Equilibrium investigation experiments were performed in triplicate to provide a clear understanding of the adsorption system.

#### 2.4.3 Effect of adsorbent dosage

Adsorbent dosage tests were performed to determine the HBP efficiency for a given initial concentration (5–50 mg·L^−1^) and studied by varying the amount of adsorbent from 1-5 g while adjusting the pH and the metal ion concentration was kept constant for a given a reaction time of 24 h at 25 ± 2 °C.

## 3 Results and discussion

### 3.1 Characterization of HBP

The IR spectra of HB_5_ were analyzed in the range 4000 cm^−1^ to 500 cm^−1^ as shown in Figure 3, with bands in the higher region observed at 3615 cm^−1^, 3411 cm^−1^, 3696 cm^−1^, 1644 cm^−1^, while the lower region bands were observed at 1436 cm^−1^, 1384 cm^−1^, 1035 cm^−1^, 914 cm^−1^, 729 cm^−1^, 518 cm^−1^ and 464 cm^−1^ featuring distinctive absorption bands at 1436 cm^-^1,1035 cm^−1^ and 518 cm^−1^ with some differences in their comparative intensities which is attributed to bentonite as well as humic's acid expansion of functional groups [23]. The bands were assigned according to Aftab et al. (2021) [23] as follows: The sharp bands observed at 3615 cm^−1^ and 3411 cm^−1^ are an indication of the presence of bentonite in HBP because band characteristics of bentonite are generally observed at 3400-3600 cm^−1^ in attribution to O–H stretching vibrations of the Si–OH group [25,26]. However, in the IR spectra of HB_5,_ there are shifts observed in these bands resulting in bands 3615 cm^−1^–3411 cm^−1^ which can be attributed to activation of bentonite. The single stretched band at 3411 cm^−1^ is owed to OH stretching of OH^−^ of water present and this proves possible hydroxyl linkages occurring between tetrahedral layers of the bentonite clay as well as minor N–H stretching of various functional groups in sodium humate [23].

A very sharp, intense, and broadband observed at 1644 cm^−1^ is due to the asymmetric OH stretching (deformation mode) of water and is a structural part of the montmorillonite mineral [23], which is what gives HBP a large surface area per unit weight function and an ion exchange capacity function. It can also be assigned to aromatic C=C skeletal vibrations, C=O stretching of quinone and amide groups (amide I band), C=O of H-bonded conjugated ketones of sodium humate [23], which is what gives a crucial impact on acidity, ion exchange properties, colloidal properties and complexing properties of HBP [23].

As reported by Aftab et al. (2021) [23], when studying the complexation properties of HA to divalent cations, the formation of metal ion complexes and the interaction between metal cation and the carboxyl groups lead to the disappearance of absorption peaks 1700 cm^−1^ 1720 cm^−1^ [23], which is similar in contrast to result obtained in characterizing HBP composite as it is observed (Figure 3).

It is therefore assumed that in this case the emergence of new bands observed at around 1583 cm^−1^ and 1384 cm^−1^, normally assigned to COO^−^ group and its stretching, are absent and the absence of this group only indicates that the majority of the COOH groups have been converted to the COO type [23]. Unlike the (COO stretch), the 1644 cm^−1^ band observed is believed to be from pure HA together with the change to lower frequencies confirming the formation of HA-M^+^, and the band around 1436 cm^−1^ (COO stretch) falls for HA-M^+^ complexes [23], signifying that humic acid-metal complexations are even more intense. In summary, it can be concluded that the complexes of humic/metal substances in HBP are mostly formed through metal-carboxylate bonds [23]. Again, in the lower region, the band observed at 1036 cm^−1^ may also be due to the vibrational mode of the SiO_4_ tetrahedron and is a feature of the layered silicate montmorillonite mineral associated with the triply degenerate Si–O stretching mode [23], which is a component of maximum adsorption band. The band observed at 913 cm^−1^ is due to the OH deformation mode of Al–Al–OH or Al–OH–Al [27].

**Figure 4:**
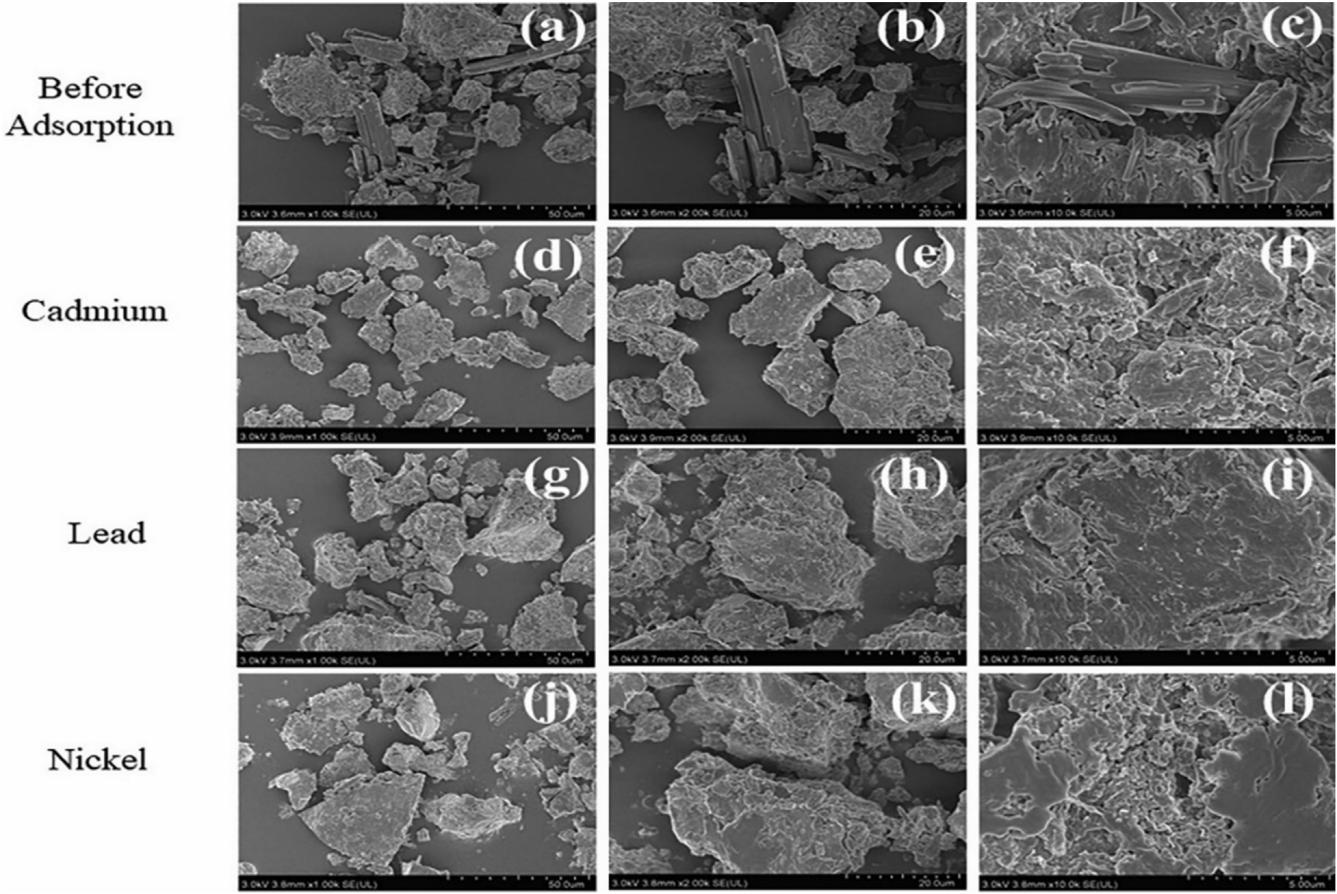
Scanning electron microscope of HBP surface change morphology before adsorption. (a); (b) and (c) HBP surface morphology before adsorption; (d); (e) and (f) HBP surface morphology after adsorption of Cd^2*+*^; (g); (h) (I); HBP surface morphology after adsorption of Pb^2+^; (j) (k); (l) HBP surface morphology after adsorption of Ni^2+^. [ experimental conditions: temperature (T) = 25 ± 2 °C, initial concentration (I) = 5 mg·L^−1^, adsorbent dosage = 1 g·L^−1^, and reaction time (t) = 24 h].

The obtained SEM characterization results in Figure 4 show evidence of bentonite, humic acid, and polymer while showing the difference between the surface morphology of the composite before and after adsorption. The original surface morphology of the HBP particles in Figure 4 (a, b, c) before adsorption shows an uneven texture due to the modified sodium bentonite and also because the micromorphology of the original bentonite particles usually presents a thick layer with an overall uneven and sporadic distribution [28]. The presence of irregular acicular growths indicates the presence of polymer. The micromorphology before adsorption has rough surfaces with an irregular concave and convex structure attributed to a porous phenomenon, and the inclusion of fine particles in some HBP channels provides adsorption sites. The presence of humic acid is evidenced by the appearance of flake-like textured formations that lead to particle distortion and fragmentation. However, after adsorption, Figure 4 (d, e, f, g, h, I, j, k, l) shows an almost absent crystallinity and smooth surface edges, which is due to the adsorption loading of the composite with Pb^2+^, Ni^2+^, and Cr^2+^ cations, indicating effects of Pb^2+^, Ni^2+^, and Cr^2+^ adsorption on HBP.

**Figure 5:**
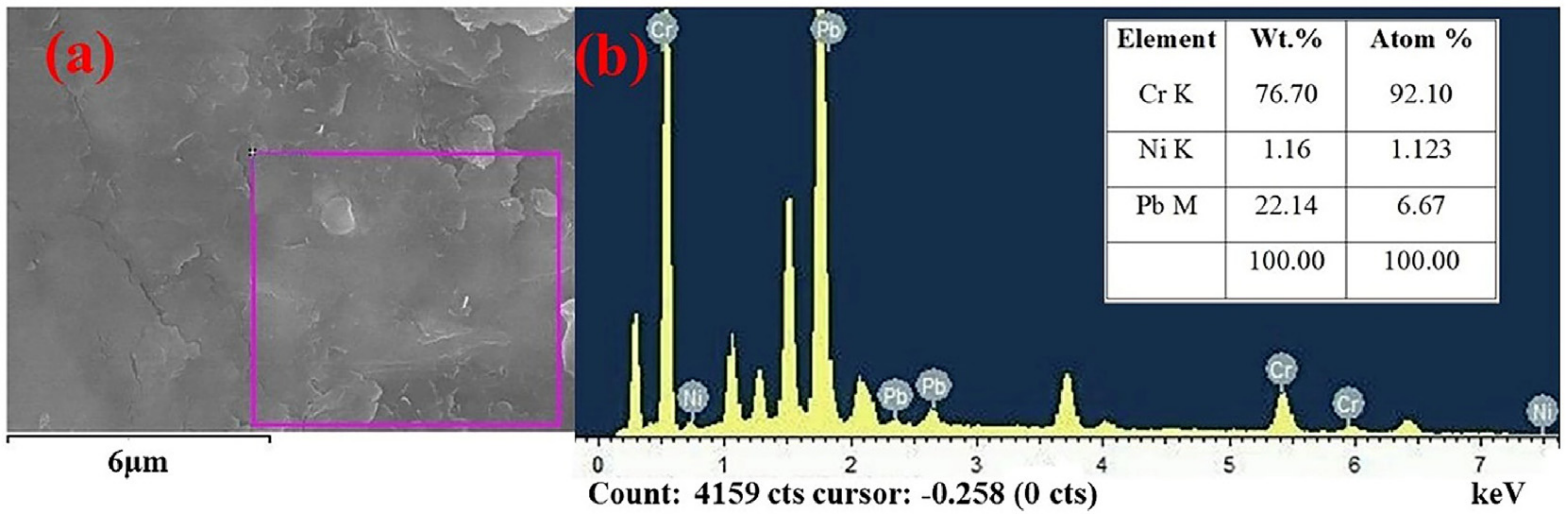
SEM-EDS analysis of HBP composite after adsorption of Pb^2+^, Ni^2+^, and Cd^2+^ from a multiple solute phase reaction. (a) SEM analysis of size distribution homogeneity of the particle size; (b) energy-dispersive X-ray of elements adsorbed on HBP.

Figure 5 below shows the morphological structure of the HBP composite after adsorption of Pb^2+^, Ni^2+^, and Cd^2+^. The SEM provides information on the size distribution homogeneity of the particle size and the energy dispersive X-ray gives insight into the elements adsorbed on HBP, their spatial distribution, and relative concentration volumes. The contrasts measured by this SEM-EDS analysis were semi-quantitative elemental analyses, therefore the reliability of the results obtained may be influenced solely by factors such as sample morphology and matrix, among other factors. Considering that the adsorption test on which these results are based was multiple solution-phase reactions, the element concentration after adsorption showed higher amounts of chromium than the other two metal ions, in addition to the atomic weights of the elements obtained, the adsorption of these three metal cations on HBP can be detected, although the results are different, the difference can be attributed to the heterogeneity distribution of the microstructure components and additionally to the fact that other components can be hidden under the sample surface, affecting the EDS results [29]. Therefore, the quantitative analysis of the elemental composition should be preceded by investigations into the homogeneity of the analyzed sample volume. Just as by scanning electron images, the EDS technique could provide images of the relative distribution of chemical element concentrations on the sample surface [29].

### 3.2 Evaluation of HBP adsorption efficiency

The sorption process of heavy metal ions is initially dependent on factors such as pH, adsorbent dosage, the influence of time, the effect of initial metal ion concentration, and some organic matter. Using the selected adsorption material with the best performance, the effectiveness of HBP was studied based on the variation of the concentration of heavy metal ions and the contact time of the process, while keeping the adsorbent dosage and pH for each metal ion constant according to the pH that gave the maximum adsorption efficiencies were kept constant during equilibrium studies.

#### 3.2.1 Effect of solution pH

**Figure 6:**
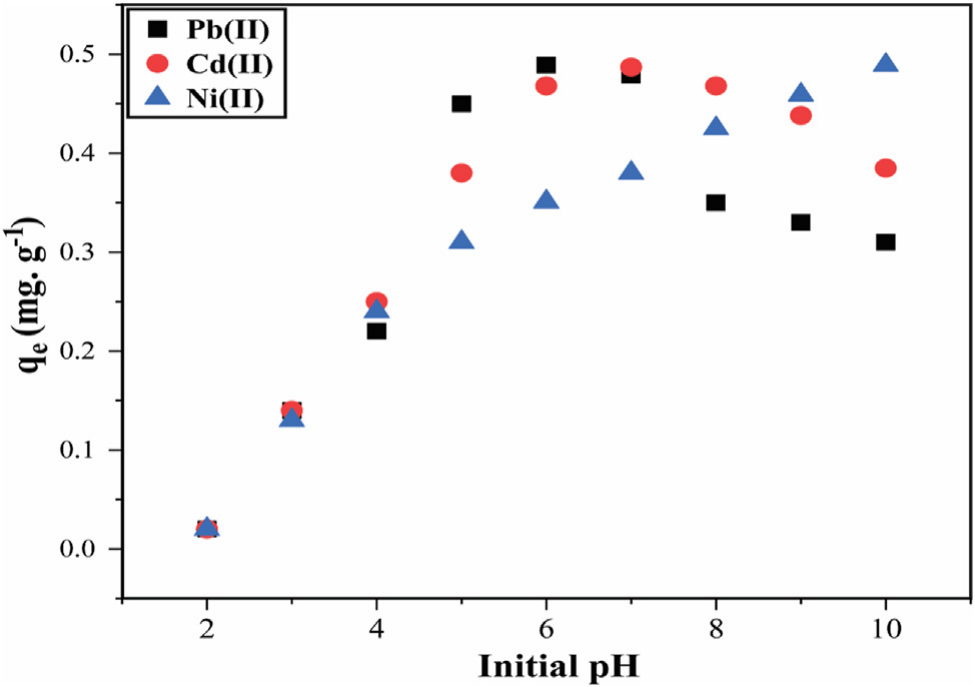
Effect of initial solution pH on the adsorption of Pb^2+^, Ni^2+^, and Cd^2+^ by HBP. [experimental conditions: temperature (T) = 25 ± 2 °C, initial concentration (I) = 5 mg·L^−1^, adsorbent dosage = 1 g·L^−1^, and reaction time (t) = 24 h].

The sorption of Pb^2+^, Ni^2+^, and Cd^2+^ by HBP is shown in Figure 6, showing a rapid increase in metal ion adsorption from a pH range of 4 to maximum uptake at pH 6, while that of Cd^2+^ shows a rapid increase in adsorption from the pH value of 4 with maximum uptakes obtained at pH 6.5. Sorption of Ni^2+^ ions similarly increased with increasing pH from pH 5 and then reached maximum uptakes at pH 9 without observing precipitation of metal complexes, which might distinguish between adsorption and precipitation of heavy metals.

In numerous adsorption systems, the adsorption of metal cations from aqueous solution is strongly pH dependent [30], this may be owing to the fact that hydrogen ions are strong competing ions, and the solution pH state influences the chemical speciation of metal ions [18,30]. When pH is in the lower acidic such as 1–3, the adsorbate is to be strongly acidic, and in this moment the adsorbent is presumed to be surrounded by hydrogen ions which re capable of preventing heavy metal ions from approaching the binding sites on the adsorbent [30]. Because of this, sorption capacity is believed to be increasing with increasing pH. The influence of solution pH on sorption is attributed to the alterations in net proton charge on particles, and at low solution pH, low to minimal sorption may be observed, owing to higher concentration as well as the high mobility of H^+^ ions which are preferentially adsorbed rather than metal ions [18]. As pH increases, so does electrostatic repulsion, thus enhancing metal cations adsorption, respectively. However, as the charge density increases, there is an electrostatic repulsion observed between heavy metal ions and positive charges on the sorption edges of the adsorbent [18]. Therefore, we can deduce that the increase in electrostatic repulsion is imputable with an increase in negative charges on the surface of sorbent, which increases adsorption of heavy metal ions [18].

#### 3.2.2 Effect of initial metal concentration on adsorption efficiency

**Figure 7:**
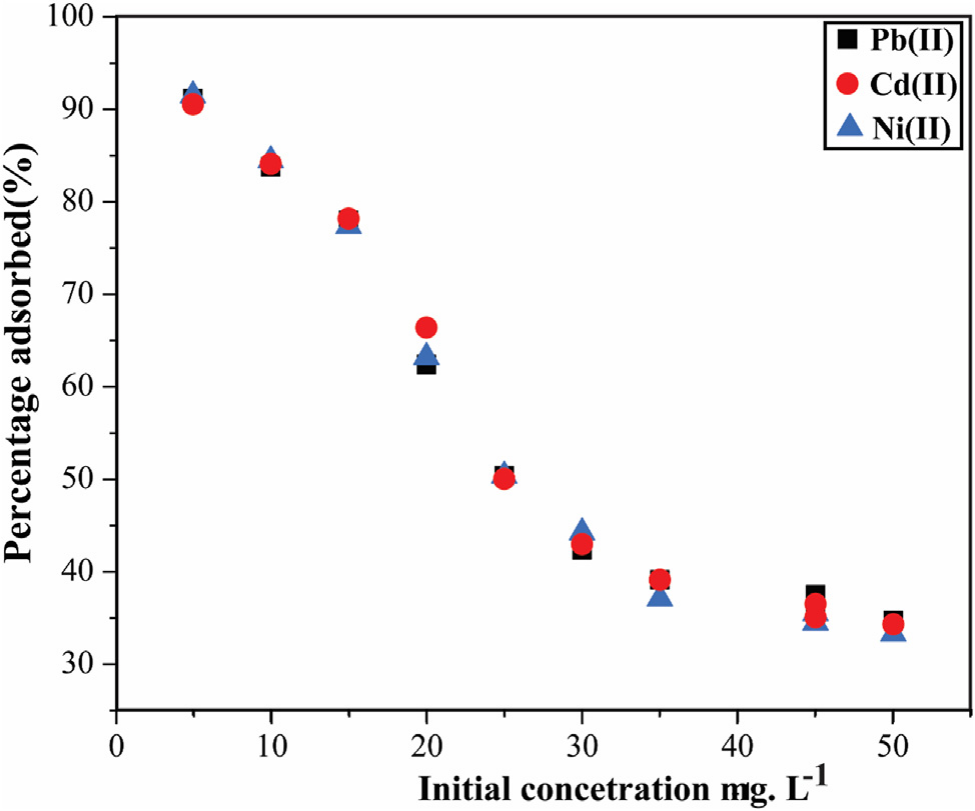
Adsorption of Pb^2+^, Ni^2+^, and Cd^2+^ onto HBP as a function of initial concentration at their respective pH. [experimental conditions: T = 25 ± 2 °C, I = 5 mg·L^−1^, adsorbent dosage = 1 g·L^−1^ and t = 24 h].

The effect of changing adsorption efficiency as a function of initial concentration is shown in Figure 7. The initial metal concentration (*I*) was varied from 5 to 50 mg·L^−1^ for Pb^2+^, Ni^2+^, and Cd^2+^ in split solutions at their respective pH values (5, 6, and 9) while keeping time and adsorbent dosage constant. The highest adsorption efficiency obtained among the three metal cations was 94.7%, 90.9%, and 90.2% in ascending order for the cations Pb^2+^, Ni^2+^, and Cd^2+^. The sorption percentage of Pb^2+^, Ni^2+^, and Cd^2+^ on HBP is calculated by the following Eq. (8) below:

(8) $$Removal\;\left(\%\right)=Co-\frac{Ct}{Co}*100$$

where Co is the initial Pb^2+^, Ni^2+^, and Cd^2+^ ion concentration (mg·L^−1^) and Ct (mg·L^−1^) is the equilibrated Pb^2+^, Ni^2+^and Cd^2+^ ion concertation at the time (*t*).

In a general adsorption system, when metal ion concentration increases, the adsorption efficiency decreases due to adsorbent saturation [24,25,30], and this gives an indication that the available adsorption sites or functional groups also increases with an increase in adsorbent, and also the perceived interactions may occur easily between the sorbent and metal ions as the sorbent amount decreases at a fixed metal ion concentration [30]. The adsorption efficiency of Pb^2+^, Ni^2+^, and Cd^2+^ onto HBP decreases with increasing initial concentration as observed in Figure 7, but the possibility that these three metal cations may be having different sorption capacities onto HBP is conceivable. While these differences maybe negligible, they can be explained by the disparity in cation radius and interaction of enthalpy values [18] or they can be explained by the fact that the sorption capacity of humic acids for metals is much higher than their possibilities as given by the extra present functional group content, and, therefore, metal ions tend to be bound to humic acids at different rates, hence the use of humic acids to remove heavy metal ions is of great potential, focusing on the (often problematic) low concentrations factor and the stability of the complexes [16]. The adsorption capacity of Pb^2+^ is slightly higher than that of Cd^2+^, which agrees with the results reported by Xu [28], and the reason of this maybe that either the diameter of Pb^2+^ is larger than that of Cd^2+^, which attracts Pb^2+^ ions by electrostatic attraction to reach the surface of the adsorbent and then ion-exchange reaction and surface hydroxyl chemical adsorption with the ions in bentonite or the greater electrical negativity of Pb^2+^ (2.33) than that of Cd^2+^ (1.69) contributed to the higher qe of Pb^2+^ than Cd^2+^, because of this, metal ions with greater electrical negativity accept electrons more easily and are absorbed [28].

The HBP adsorption results demonstrated that as the metal ion concentration in the solution is increased, the concentration disparity between the bulk solution and the surface also increased, intensifying the mass transfer processes [18,34]. HBP surface saturation is thought to be one of its adsorption properties and is dependent on the metal ion concentration, with HBP active sites rapidly taking up most of the available metal ions at low concentrations, whereas at higher concentrations metal ions are expected to diffuse to the sorbent surface through intraparticle diffusion and heavily hydrolyzed ions tend to diffuse more slowly [18]. These occurrences could be attributed to several factors such as: (1) at low initial heavy metal ion concentrations, there is plenty available free pores, and binding sites on HBP sorbent, hence the fractional adsorption and mass transfer of heavy metal ions are high [35]; resulting into high percentage removals at initial concentrations below 15 mg·L^−1^, (2) as the initial concentration increases from 20 mg·L^−1^ to 35 mg·L^−1^, the mass transfer force of heavy metal ion decreases, resulting in a low adsorption percentage onto the remaining available binding sites [35], and (3) as the initial concentration increases further above 35 mg·L^−1^ and especially at 50 mg·L^−1^, the ratio of heavy metal ions to available binding sites is at levels that do not support mass transfer [33]. It can therefore be inferred that the HBP adsorption is a three-stage process, beginning with:

i. mass transfer step of heavy metal ions from the adsorbate bulk to the surface of HBP particles through the boundary layer around HBP.
ii. Diffusion step where internal diffusion of Pb^2+^, Cd^2+^, and Ni^2+^ through the HBP adsorbent pores takes place.
iii. Adsorption step where adsorption of Pb^2+^, Cd^2+^, and Ni^2+^ onto the surface of the HBP takes place.

In brief, metal ions were initially diffused from the boundary layer film onto the adsorbent surface and finally diffuse into the porous structure of the sorbent [13,17].

#### 3.2.3 Effect of contact time on q_e_

**Figure 8:**
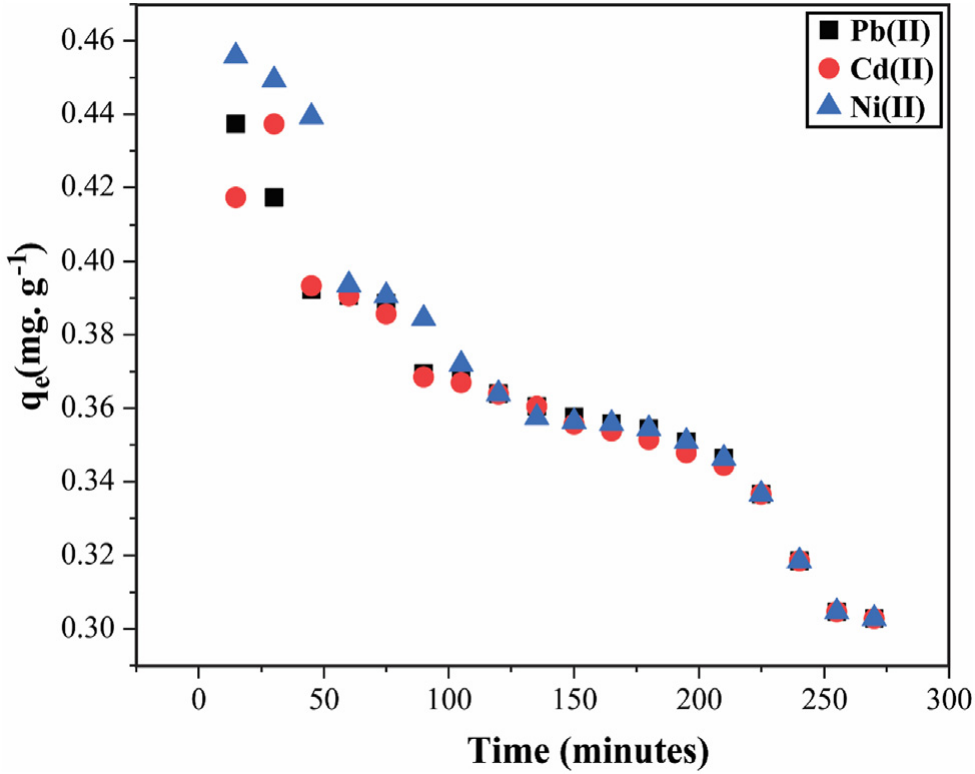
Effect of contact time on q_e_ ((maximum amount adsorbed onto HBP at the time (t)) for the adsorption of Pb^2+^, Cd^2+^, and Ni^2+^ by HBP. [experimental conditions: T = 25 ± 2 °C, I = 5 mg·L^−1^, adsorbent dosage = 1 g·L^−1^ and t = 5 h at their respective pH].

The adsorption kinetics of Pb^2+^, Cd^2+^, and Ni^2+^ by HBP was carried out at a contact time ranging from 15-510 min and a concentration of 5 mg·L^−1^. Figure 8 shows the amount of Pb^2+^ adsorbed by HBP for the first 300 min of the sorption process. Adsorption of Pb^2+^, Cd^2+^, and Ni^2+^ by HBP appears to be a three-step process. The removal of heavy metal ions increases sharply during the first 10–30 min, with the number of heavy metal ions adsorbed per unit mass reaching 0.45 mg·g^−1^, 0.44 mg·g^−1^, and 0.43 mg·g^−1^ at an initial concentration of 5 mg·g^−1^.

The increase in *q*_*e*_ (maximum amount adsorbed on HBP at a time *(t*)) therefore decreases more slowly with increasing time and began to decrease significantly after 1 h, when assumed equilibrium was reached, and later followed by HBP saturation where no adsorption was observed at 500 min and above. It can be concluded that in this study the fast adsorption was due to physically passive adsorption or ion exchange at the absorbent surface, respectively. Because there are a certain number of adsorption sites on the surface of the adsorbent, heavy metal adsorption on the adsorbent occurs rapidly at first, but over time, the number of available active sites decreases, and competition from the remaining metal ions reduces the adsorption rate due to decreased binding sites [36].

In a normal adsorption system, a maximum duration of 1 h is effective to achieve effective adsorption results, with the increase in qe rapidly decreasing and the adsorption stopping very quickly, in terms of HPB, the adsorption duration went up to 3 h with metal ion uptake is still going on observed and this can only be attributed to the unique properties of HBP complemented by both bentonite and humic acid. Because there were no competing ions in the aqueous solution, the single sorption of three different metal cations onto HBP lasted more than 60 min, unlike in a normal adsorption system, since in this single sorption the simultaneous sorption is believed to be due to two mechanisms. The first mechanism is the surface reaction by ion exchange of interlamellar cations of bentonite clays, where the formation of inner-sphere complexes with Si–O and Al–O groups at the edges [19,27] takes place, and the second B By mass transfer, where the complexation of these heavy metal ions with humic acid takes place.

#### 3.2.4 Effect of adsorbent dosage

**Figure 9:**
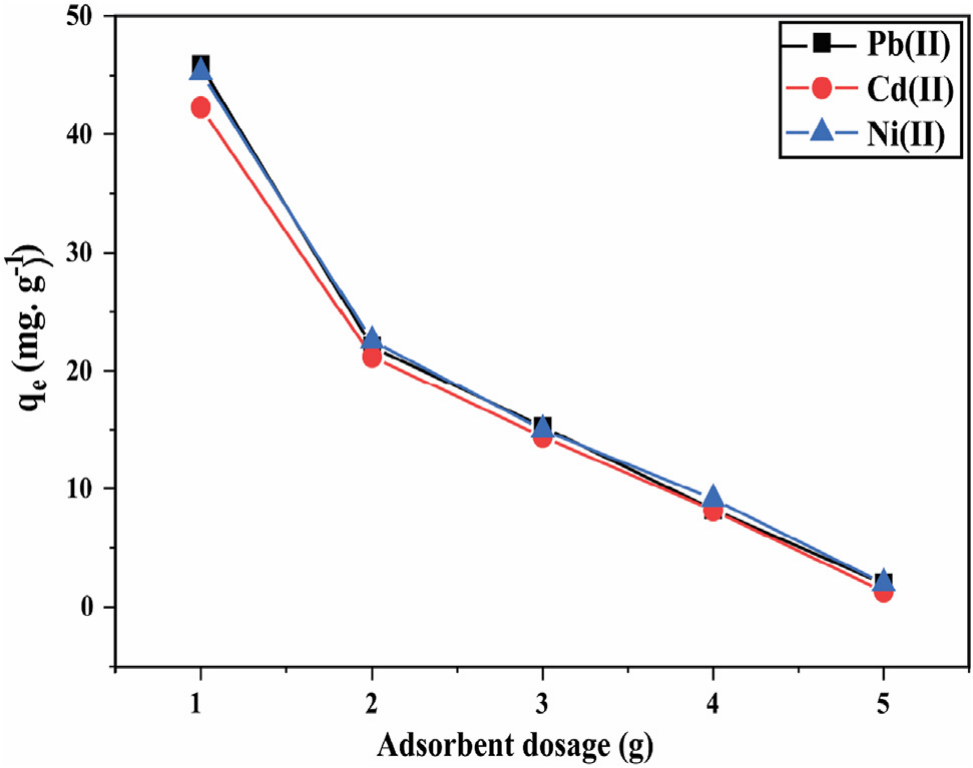
Effect of adsorbent dosage on qe for the adsorption of Pb^2+^, Cd^2+^, and Ni^2+^ by HBP adsorbent. [experimental conditions: T = 25 ± 2 °C, I = 5 mg·L^−1^, t = 24 h at their respective pH].

The effect of adsorbent dosage on the removal of Pb^2+^, Cd^2+^, and Ni^2+^ by HBP and adsorption capacity was studied and presented in Figure 9.

To determine any adsorbent's effectiveness for a given initial adsorbate concentration, adsorbent dosage is a critical factor of evaluation. The number of heavy metal ions (qe) adsorbed per adsorbent mass or the adsorption capacity decreased with increasing adsorbent dosage of 1–5 g·L^−^. As the adsorbent dosage is increased while holding all other reaction variables constant, the removal efficiency first increases and, after reaching equilibrium, later decreases at a slower slope, beginning with an increment of 2 g. Maximum adsorption capacity and highest adsorption efficiency by HBP at concentrations as low as 5 mg·g^−1^ and as high as 50 mg·g^−1^ were obtained with the adsorbent dosage of 1 g adsorbent in 100 ml deionized water.

#### 3.2.5 Effect of humic acid on bentonite content on adsorption efficiency

**Figure 10:**
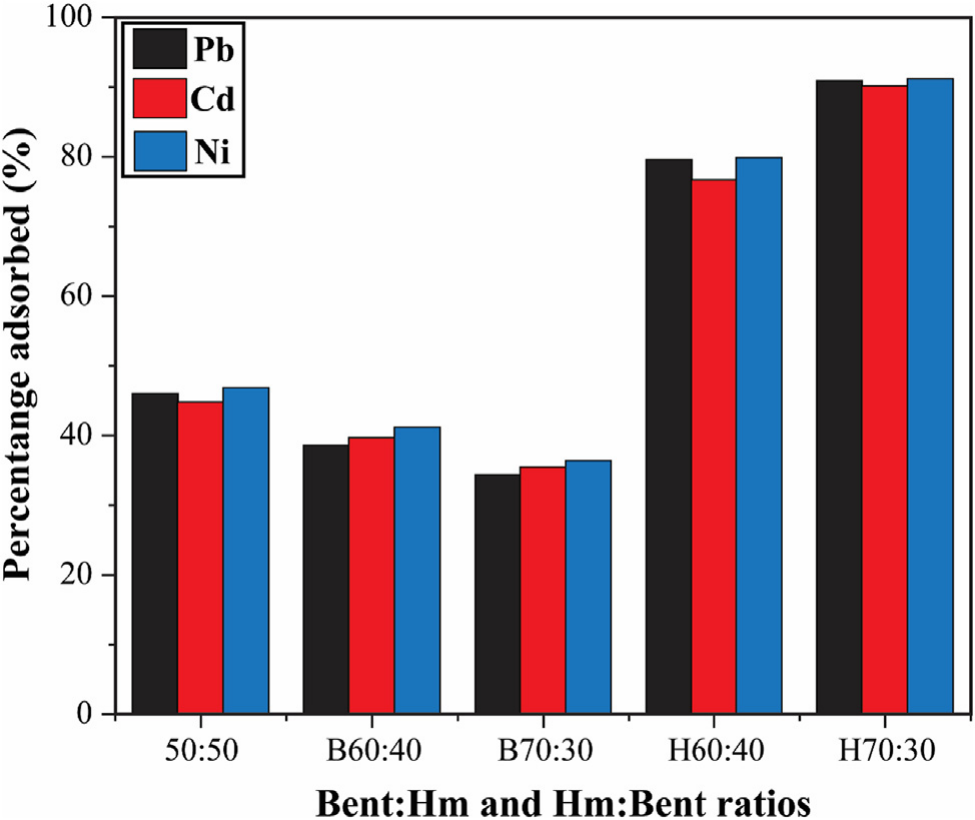
Effect of bentonite to humic acid weight ratio and vice-versa on adsorption of Pb^2+^, Ni^2+^, and Cd^2+^ by HBP adsorbent. [experimental conditions: T = 25 ± 2 °C, I = 5 mg·L^−1^, t = 24 h at their respective pH].

The sorption of Pb^2+^, Cd^2+^, and Ni^2+^ cations by HBP as a function of humic acid and bentonite content in HBP pellets is shown in Figure 10. It can be observed that the sorption percentage of both Pb^2+^, Cd^2+^, and Ni^2+^ cations increase with increasing humic acid content and a reasonable amount of bentonite in the adsorbent. Equating humic acid and bentonite content resulted in an adsorption efficiency of less than 50%, meaning that the efficiency of this adsorbent can only be increased by increasing one of the two constituent materials without completely abandoning the other constituent. Considering that cadmium showed a less low adsorption trend on HBP compared to the other two, a similar result was obtained by Martina and others [16], consistent with the fact that different metal ions have different affinities for humic acids, and they further discussed that the solubility of metal–humic complexes decrease with increasing metal: humic ratio, which might be related to the gradual decrease in the free ionized functional group.

In contrast to results found by Xiaoying [27] in the preparation of enhanced bentonite modified with humic acid (HAB) composite for the sorption of copper ions, the increase in adsorption with an increased amount of humic acid in the adsorbent could be attributed to the increase in hydroxyl and carboxyl groups composed of bentonite and humic acid, which enhances the substitution of hydrogen atoms with metal ions, giving HBP high chemical activity after interaction with heavy metal ions, complexation takes place between humic acid and heavy metal ions where chelates or coordinate bonds are formed, resulting in increased heavy metal ion uptake capacity of HBP.

The balanced functional groups responsible for maximum adsorption in HBP give the adsorbent increased heavy metal ion affinity property, but also a balanced amount of bentonite promotes ion exchange. The details are further explained by the FTIR analysis in Figure 3. Although the adsorption mechanism is mainly subsidized by chemical interactions between metal ions and surface functional groups of HBP, the content of bentonite versus humic acid is extremely significant. The presence of crucial bands responsible for promoting adsorption and increasing the affinity of heavy metal ions in HBP is dominated by the levels of sodium humate and sodium bentonite present in the adsorbent. The asymmetric OH^−^ stretching of water is a structural part of the montmorillonite mineral that gives HBP a high surface area per unit weight and a capacity for a base exchange function.

Chemical bonds between metal ions and the surface functional groups of HBP are mainly responsible for the adsorption, therefore a certain quantitative, homogeneous mixture of sodium humate and bentonite plays an important role. However, when the amount of humic acid is further increased, the adsorbent pellets have poor mechanical properties, and the adsorbent is unable to complete the adsorption reaction speed, resulting in a poor structural deformation. This may be due to an insufficient contribution to the colloidal properties of bentonite and itself being a natural binder that also contributes to the mechanical properties and strength of the pellets. This leads to poor absorption or adsorption reduction, which can also be attributed to insufficient ion-exchange base capacity provided by bentonite or blocking of bentonite pores by the polymer since the content of humic acid in the pellets requires somewhat more polymer addition.

### 3.3 Evaluation of adsorption isotherms

To evaluate the sorption mechanisms of heavy metal ions by HBP, Langmuir and Freundlich isotherm models are applied to describe the sorption data in this study. The constants and correlation coefficients of Pb^2+^, Cd^2+^, and Ni^2+^ cations adsorption by HBP are presented in Table 4.

**Table 4:** Isotherm parameters for sorption of Pb^2+^, Cd^2+^ and Ni^2+^ cations ions by HBP adsorbent.

Isotherm Model	Pb^2+^	Ni^2+^	Cd^2+^
***Langmuir***			
*q*_*max(mg/g)*_	2.8950	2.6170	2.6136
*K*_*L(L/mg)*_	0.1942	0.3532	0.1959
*R*_*L*_	0.0289	0.7660	0.02034
*R*^*2*^	0.9844	0.98013	0.98366
***Freundlich***			
*R*^*2*^	0.93428	0.92428	0.99304
*1/n*	0.32777	0.35749	0.26152
K_f_	0.39587	0.38558	0.40460

The correlation coefficient (*R*^*2*^) of Pb^2+^ by Langmuir isotherm was 0.9844 and higher than that of Freundlich isotherm, 0.93428. In short, it is closer to the value of 1 compared to that of the Freundlich isotherm, the same applies to Ni^2+^, which suggests that the adsorption of the two metal cations onto HBP favors the Langmuir Isotherm model, implying monolayer adsorption with the energy of the adsorbent being the same on the adsorbent surface and adsorbed links assumed to be reversible, however, this does not mean the adsorption process of Pb^2+^, Cd^2+^, and Ni^2+^ ions by HBP entirely follows this model, as the adsorption process is complex and may follow several isotherms in general, respectively. The dimensionless constant separation factor (R_*L*_) for the two metal cations gave values of 0.0289 and 0.7660 that are less than 1, indicating a favorable isotherm shape respectively. Cadmium conversely presented results that favor Freundlich isotherm over Langmuir isotherm with a negligible difference and the correlation coefficients of the two isotherms being 0.99304 and 0.98366. In a Freundlich isotherm model, multilayer adsorption on heterogeneous surfaces where interactions between adsorbed molecules take place. Here the adsorbent surfaces are considered not to be uniform and the adsorption force differs. The vital characteristics of Freundlich isotherm are characterized by the value of 1/n, which ranges between 0 and 1 and is a measure of adsorption intensity or surface heterogeneity, which indicates more heterogeneity as its value gets closer to zero. However, this does not entirely mean adsorption of Cd^2+^onto HBP by Langmuir is not favorable and entirely follows the Freundlich model, as the adsorption process is complex and may follow several isotherms, in general, depending on the characteristics of the adsorbent.

**Figure 11:**
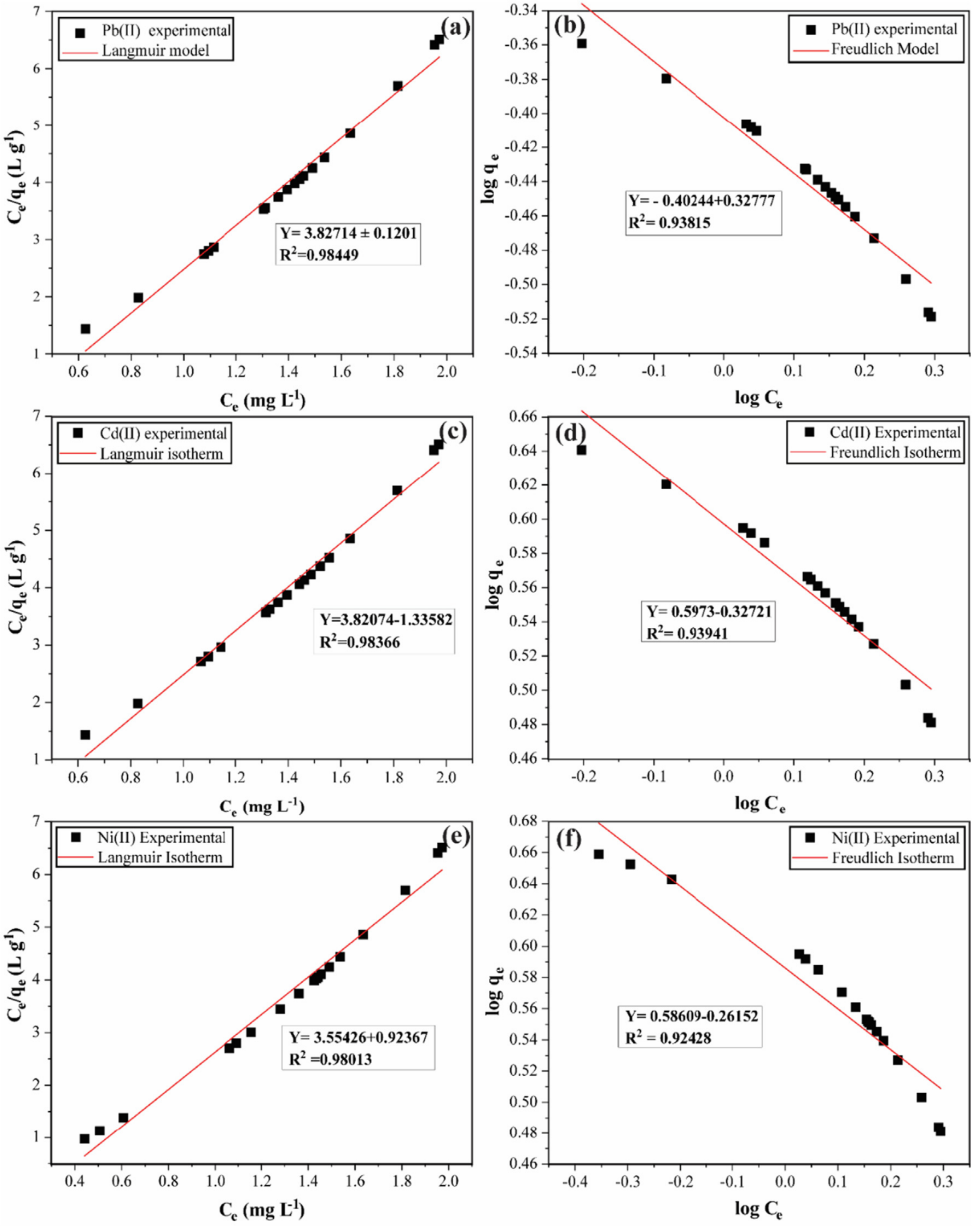
(a–f). Adsorption isotherms of Pb^2+^, Cd^2+^, and Ni^2+^ cations by HBP. [experimental conditions: T = 25 ± 2 °C, I = 5 mg·L^−1^, t = 24 h at their respective pH].

The plots for Langmuir and Freundlich isotherms for both heavy metal ions are presented in Figure 11.

#### 3.4 HBP adsorption efficiency vs other bentonite and humic acid-containing adsorbents

The investigation of HBP adsorption efficiency compared to other composites constituting humic acid, bentonite, and polymeric materials is presented in Table 5. Disposing of other effects such as initial concentration, contact time, and adsorbent dosage, the comparison was mostly made upon adsorbent constituents complementing the sorption process such as bentonite's cation exchange capacity, and humic acid's ability to adsorb and complex-ate with heavy metal ions.

**Table 5:** Comparison of HBP adsorption efficiency vs other bentonite and humic acid constituting adsorbents.

Adsorbent	Metal ions	Qe (mg·g^−1^)	Concertation (mg·L^−1^)	pH	*R*^*2*^	Isotherm	Reference
Humic acid-Bentonite -Polymer Composite (HBP)	Pb^2+^	4.570	5	5.0	0.9844	Langmuir	Present study
	Cd^2+^			6.0	0.9930	Freundlich	
	Ni^2+^			9.0	0.9801	Langmuir	
Polyacrylamide-polystyrene/Bentonite nanocomposite	Cd^2+^, Pb^2+^	8.04	10	2–7	0.9474	Langmuir	[37]
Humic acid immobilized Polymer-Bentonite composite (HA-Am-PAA-B)	Cu^2+^	97.9	200	5.0	0.998	Langmuir	[38]
				6.0	0.998	Freundlich, D-R isotherms	
Modified Bentonite with Humic acid (HAB)	Cu^2+^	22.40	100	6.5	–	–	[27]
Super absorbent Bentonite-Polyacrylamide composite	Cu^2+^	50.07	100	5.0	0.9924	Langmuir	[8]
				6.2	0.9827	Langmuir	

Generally, it is expected that competition between H_2_O_3_ M^2+^ ions reduce adsorption capacity at a low pH, [37], therefore in the higher acidic regions, preferably at pH values ranging from 4-7 most adsorbents perform better in comparison to their performances in lower acidic regions. As the adsorption extent is withered in lower acidic regions, increasing pH thereby increases adsorption, and this is one of the dependent variables in adsorption affecting the efficiency of many adsorbents. Mahdavi et al. [37], prepared a Polyacrylamide-polystyrene/Bentonite nanocomposite for immobilizing Cd^2+^ and Pb^2+^ions in the soil, and in this study, he discusses the adsorption efficiency of the nanocomposite in comparison to polyacrylamide or bentonite alone, which was found to be quite better, hereby signifying the presence of multiple material properties in a single adsorbent resulting in enhanced adsorption capacity [37]. Their results showed that the adsorption of Cd^2+^ and Pb^2+^ ions by bentonite occurs happens via cation exchange, and even though the base exchange ability of bentonite is limited, there is again adsorption of these metal ions by the polymer which takes place via the interactions existing between the Oxygen and Nitrogen functional groups of the polymer and adsorbate ion [37]. Again, these however, these relations plus those of bentonite maybe and or are limited, and could not possibly aid a successful removal of Cd^2+^ and Pb^2+^ ions from the sorbate, hence the room for innovation of such composites to improve their selectivity and adsorption properties. The distinct polyacrylamide-polystyrene/bentonite nanocomposite properties are the fact that it is able to enjoys both the ion exchange abilities of bentonite and the interactivity between N and O groups of the polymer and the cations [37], therefore the scope of adsorption by the nanocomposite was substantially higher.

HBP sorption results obtained in contrast to polyacrylamide-polystyrene/bentonite nanocomposite are presented in Table 5. A composite complemented by both polymer, bentonite, and humic acid adsorption properties is indeed modest. This is attributed to the fact that in HBP, the adsorption effectivity is complemented by the cation exchange property of bentonite and its surface properties, and with the limitations that comes with the cation exchange capacity of bentonite, the additional adsorption effectivity owed to polymer fibers by the functional polymer takes place through the interaction between them contributing to the oxygen and Nitrogen groups provided by aPAM and the adsorbate ion. However, this sorption property is also limited, and humic acid function still has room to play its role, thereby enhancing adsorption with its ability to sorb cationic pollutants and high affinity for heavy metals, consistent with observations in the literature [10,28].

HBP thereby enjoys both the ion exchange potentials of bentonite, the interaction between nitrogen and oxygen groups of the polymer, and the high affinity of humic acid for heavy metal ions, resulting in enhanced adsorption capacity. Xiaoying et al. [27], characterized bentonite modified with humic acid into a single adsorbent by modifying bentonite with humic acid to obtain novel adsorbent, bentonite modified with humic acid (HAB) for the simultaneous removal of Cu^2+^ and 2,4-dichlorophenol (2,4-DCP) from aqueous solution [27]. The results showed that HAB slightly enhances the sorption of Cu^2+^ with almost 23 mg·g^−1^ Cu^2+^ adsorbed which is 1 mg·g^−1^ more than unmodified bentonite (UB) alone. This indicates the complexation existing between HA and metal ions on the surface of bentonite, although these complexation processes are usually very fast in complexation studies of HA [27]. They further suggested that, the sorption of Cu^2+^ onto HAB may be owed to the ion exchange mechanism moderately than the complex formation mechanism [27]. However, the results for initial sorption of 2,4-DCP onto both UB and HAB were rapid, and he highest adsorption efficiency being that of 2,4-DCP onto HAB, and this could be attributed to the interaction between 2,4-DCP and HA on the surface of HAB., In addition, HA was one of the main components present in the composite which means additional functional properties such as hydrophilicity and hydrophobicity of molecules in the composite. Lastly, the hydrophobic interactions occurring between HA and 2,4-DCP are believed to have enhanced the sorption of M^2+^ ions [27].

## 4 Conclusion

Based on the obtained HBP adsorption efficiency, the results of adsorption kinetics, the properties of the HBP composition, and the observed functional groups present, we can draw the following conclusions:

• The synthesis of HBP is part of the ongoing effort to develop improved adsorbents in the wastewater treatment industry that support the use of naturally abundant and non-toxic adsorbents to characterize the effectiveness of coupling material properties. In this study, the performance of HBP for uptake of Pb^2+^, Cd^2+^, Ni^2+^ Pb2+, Cd^2+^, and Ni^2+^ was investigated by several fixed-bed adsorption experiments.
• HBP mainly contained functional groups of Si–OH, Al–Al–OH, N–H, and C=O to which the effectivity of HBP is owed.
• The obtained adsorption results showed that the sorption of Pb^2+^, Cd^2+^, and Ni^2+^ Pb^2+^, Cd^2+^ and Ni^2+^ ions by HBP strongly depends on pH, initial concentration, and surface complexation between adsorbent and adsorbate, which is complemented by the properties of humic acid and bentonite.
• HBP adsorption efficiency in removing Pb^2+^, Cd^2+^, and Ni^2+^ ions at the respective pH values of 5, 6.5, and 9 was outstanding, giving high adsorption efficiencies in ascending order for the cations Pb^2+^, Ni^2+^, and Cd^2+^ of 94.7%, 90.9%, and 90.2%, while the maximum amount of metal cations adsorbed per unit mass (q_e_) at the lowest initial concentration (5 mg·L^−1^) was 0.457 mg·g^−1^, 0.442 mg·g^−1^ and 0.43 mg·g^−1^.
• The adsorption data obtained fitted Langmuir isotherm for Pb^2+^and Ni^2+^, with the correlation coefficients of (*R*^*2*^ = 0.9844 and 0.9813) while the adsorption for Cadmium fitted the Freundlich isotherm model better *(R*^*2*^ = 0.99304). The monolayer adsorption capacity (q_max_), obtained from Langmuir isotherm for Pb^2+^and Ni^2+^ was 2.895 mg·g^−1^ and 2.6170 mg·g^−1^ while that of Cadmium was 2.6136 mg·g^−1^.
• Compared to composites of bentonite, humic acid, and polymers, HBP composite has shown higher adsorption efficiency as well as adsorption capacity, making it a suitable adsorbent in wastewater treatment for removing heavy metal ions.
• The synthesis of HBP is of low cost and can be accomplished by constructing an appropriate column and using HBP pellets as the adsorbent, but further research is hereby necessary to explore HBP desorption, regeneration, adsorbent recyclability, and to examine its actual application performance in wastewater treatment.
• The complexes of humic/metal substances are formed predominantly by metal-carboxylate bonds.

## Declarations

### Author contribution statement

Evelina L.M Amutenya: Conceived and designed the experiments; Performed the experiments; Analyzed and interpreted the data; Wrote the paper.

Fengshan Zhou: Conceived and designed the experiments; Contributed reagents, materials, analysis tools or data.

Jinliang Liu, Wenjun Long, Liang Ma: Analyzed and interpreted the data.

Meng Liu: Performed the experiments.

Guocheng Lv: Contributed reagents, materials, analysis tools or data.

### Funding statement

This work was supported by Fundamental Research Funds for Central Universities(No. 2-9-2019-141).

### Data availability statement

Data included in article/supplementary material/referenced in article.

### Declaration of interests statement

The authors declare no conflict of interest.

### Additional information

No additional information is available for this paper.
